# *Bifidobacterium longum* K5 Prevents Enterohaemorrhagic *Escherichia coli* O157:H7 Infection in Mice through the Modulation of the Gut Microbiota

**DOI:** 10.3390/nu16081164

**Published:** 2024-04-13

**Authors:** Deyu Liu, Chunyan Li, Ting Cao, Xiuli Lv, Yingxue Yue, Shuang Li, Yang Cheng, Fei Liu, Guicheng Huo, Bailiang Li

**Affiliations:** 1 Key Laboratory of Dairy Science, Ministry of Education, Northeast Agricultural University, Harbin 150030, China; b211001004@neau.edu.cn (D.L.); licy0005@163.com (C.L.); cao18088783239@163.com (T.C.); l04270227@163.com (X.L.); 13091442027@163.com (Y.Y.); lis202305@163.com (S.L.); m18249626875@163.com (Y.C.); david.as@163.com (F.L.); 15846092362@163.com (B.L.); 2 Food College, Northeast Agricultural University, Harbin 150030, China

**Keywords:** *Bifidobacterium longum*, enterohemorrhagic *Escherichia coli*, inflammation, gut barrier, gut microbiota

## Abstract

Enterohemorrhagic *Escherichia coli* (EHEC) serotype O157:H7 is a commonly encountered foodborne pathogen that can cause hemorrhagic enteritis and lead to hemolytic uremic syndrome (HUS) in severe cases. *Bifidobacterium* is a beneficial bacterium that naturally exists in the human gut and plays a vital role in maintaining a healthy balance in the gut microbiota. This study investigated the protective effects of *B. longum* K5 in a mouse model of EHEC O157:H7 infection. The results indicated that pretreatment with *B. longum* K5 mitigated the clinical symptoms of EHEC O157:H7 infection and attenuated the increase in myeloperoxidase (MPO) activity in the colon of the mice. In comparison to the model group, elevated serum D-lactic acid concentrations and diamine oxidase (DAO) levels were prevented in the K5-EHEC group of mice. The reduced mRNA expression of tight junction proteins (ZO-1, Occludin, and Claudin-1) and mucin MUC2, as well as the elevated expression of virulence factors Stx1A and Stx2A, was alleviated in the colon of both the K5-PBS and K5-EHEC groups. Additionally, the increase in the inflammatory cytokine levels of TNF-α and IL-1β was inhibited and the production of IL-4 and IL-10 was promoted in the K5-EHEC group compared with the model group. *B. longum* K5 significantly prevented the reduction in the abundance and diversity of mouse gut microorganisms induced by EHEC O157:H7 infection, including blocking the decrease in the relative abundance of *Roseburia, Lactobacillus,* and *Oscillibacter.* Meanwhile, the intervention with *B. longum* K5 promoted the production of acetic acid and butyric acid in the gut. This study provides insights into the use of *B. longum* K5 for developing probiotic formulations to prevent intestinal diseases caused by pathogenic bacterial infections.

## 1. Introduction

*Escherichia coli* is a prevalent bacterial species found in the human intestinal tract, which plays a vital role in food digestion. However, certain pathogenic strains can lead to severe illnesses [1]. EHEC is responsible for foodborne epidemics [2,3]. EHEC O157:H7, the most prevalent strain of EHEC, has caused numerous fatalities worldwide. Antibiotics are commonly used to treat such diseases in clinical practice. But Shiga toxin (Stx) might be induced when EHEC is treated with antibiotics, resulting in fatal disease. Moreover, the use of antibiotics may disrupt the dynamic equilibrium of the gut microbiota and contribute to increased drug resistance among pathogenic microorganisms, posing a risk of exacerbating the severity of the condition. Consequently, there is an urgent need for effective preventive measures against EHEC O157:H7 infections [4].

*Bifidobacteria*, Gram-positive prokaryotes commonly inhabiting the human gastrointestinal tract, demonstrate valuable functions [5]. Supplementation with *Bifidobacteria* can regulate the balance of the gut microbiota and prevent intestinal diseases and pathogen infections [6]. In addition, *Bifidobacteria* can inhibit the adhesion of pathogenic bacteria to intestinal epithelial cells, suppress the expression of virulence factors in different pathogens, and impede the proliferation of pathogenic bacteria through the production of metabolites such as SCFAs and bacteriocins [7,8,9]. *B. longum* is one species of *Bifidobacterium* which is prevalent in breast-fed infants and the adult intestinal tract [10]. Recent studies have emphasized the inhibitory impact of *B. longum* on EHEC O157:H7. Specifically, *B. longum* NCC2705 has been shown to offer protection against potentially fatal EHEC O157:H7 infection. It achieves this by effectively suppressing the production of Stx within the cecum and preventing its translocation from the intestinal cavity into the bloodstream [11].

The adhesion capacity is a critical parameter for assessing the potential of probiotics [12]. Probiotics with a high adhesion capacity may exert antagonistic effects on pathogens by outcompeting them, producing antimicrobial compounds, and limiting the adhesion of pathogenic microorganisms [13]. Our previous investigation indicated that *B. longum* K5 had enhanced adhesion capability on HT-29 cells and had the potential to alleviate symptoms associated with inflammatory bowel disease (IBD) [14,15]. In this study, BALB/c mice were supplemented with *B. longum* K5 for one week prior to establishing the EHEC O157:H7-induced mouse model. The expression of tight junction proteins, inflammatory cytokines, and virulence factors in the colon of the mice was examined to confirm the protective effect of *B. longum* K5 against EHEC O157:H7 infection. Additionally, to investigate the association between the gut microbiota and short-chain fatty acids (SCFAs), both the gut microbiota and SCFA concentrations were detected.

The main objective of this study was to uncover the protective mechanisms and the impacts of *B. longum* K5 against EHEC O157:H7 infection in mice. The findings of this study will establish a theoretical foundation for the development of probiotic formulations containing *B. longum* K5 as a preventive measure against EHEC O157:H7 infection.

## 2. Materials and Methods

### 2.1. Bacterial Strains

*B. longum* K5 was obtained from the Key Laboratory of Dairy Science (KLDS) at Northeast Agricultural University (NEAU) in Harbin, China. The EHEC O157:H7 strain was sourced from the China Center of Industrial Culture Collection (CICC).

### 2.2. Animals and Experimental Design

Thirty-six six-week-old BALB/c male mice were obtained from Beijing Vital River Laboratory Animal Technology Co., Ltd. (Beijing, China). They were housed under controlled conditions with a temperature of 25 ± 2 °C, a humidity of 50 ± 5%, and a 12 h light/dark cycle. The animal experiment received ethical approval from the Animal Ethics Committee of Northeast Agricultural University, with the ethical approval code NEAUEC20220363. All the mice were acclimatized for 7 days before the experiment and randomly divided into 4 groups. The experimental groups of mice are described in Table 1. From day 1 to day 7, 0.2 mL of PBS was given to the control and model groups, and 0.2 mL of *B. longum* K5 was given to the K5-EHEC and K5-PBS groups. From day 8 to day 14, 0.2 mL of PBS was given to the control and K5-PBS groups, and 0.2 mL of EHEC O157:H7 was given to the model and K5-EHEC groups. It was worth mentioning that the concentration of EHEC O157:H7 was adjusted to 1.0 × 10^8^ CFU/mL, while *B. longum* K5 was adjusted to a concentration of 1.0 × 10^9^ CFU/mL for gavage.

### 2.3. Collection of Samples

The weights of the mice were recorded before euthanasia. Immediately following euthanasia, blood samples were obtained by retro-orbital bleeding. The colons of the mice were extracted and measured for their length, and intestinal contents from all mice were collected. All the collected samples were stored at −80 °C for the subsequent experiments.

### 2.4. Disease Activity Index (DAI) Score

The mice were weighed every two days, and the DAI score was calculated based on their weight changes, fecal consistency, and bloody stools, following the criteria outlined in Table S1.

### 2.5. Determination of MPO Activity

Colonic MPO activity in the mice was measured following the instructions provided in the ELISA kit (Chenglin, Beijing), as detailed in reference [16].

### 2.6. H&E and AB-PAS Staining

Hematoxylin and eosin (H&E) staining was conducted for histological evaluation after euthanizing the mice, as described in reference [17], and the scores were computed according to the criteria provided in Table S2.

Alcian Blue/Periodic Acid Schiff (AB-PAS) staining was conducted according to the kit instructions to assess the extent of damage to the colonic mucous layer, the samples were examined under a microscope at 100× magnification, and images were captured. We quantified goblet cells in the visual field using Image-Pro Plus 6.0 software.

### 2.7. Quantitative RT-PCR

We evaluated the expression levels of tight junction proteins (ZO-1, Occludin, and Claudin-1), mucins (MUC2) in colon tissue, and virulence factors (Stx1A and Stx2A) in cecal contents using RT-qPCR, following the method described in reference [18]. The total RNA was extracted from the cells according to the method provided by the kit, and cDNA was synthesized using the reverse transcription kit. Then, RT-qPCR detection was performed using HotStart™ 2X SYBR Green qPCR Master Mix (APExBIO, Houston, TX, USA). The primer details can be found in Table S3. The relative expressions of the target genes were analyzed using the 2^−ΔΔCT^ calculation method, and Gapdh was used as the internal reference gene.

### 2.8. Detection of Gut Microbiota

DNA from each group of mouse colon contents was extracted using Fast DNA SPIN extraction kits (MP Biomedicals, Santa Ana, CA, USA). The region V3–V4 of the 16S rRNA gene was amplified with 338F/806R primers. Sequencing was performed using the Illumina MiSeq platform with MiSeq Reagent Kit v3 at Shanghai Personal Biotechnology Co., Ltd. (Shanghai, China) [19]. We employed PICRUSt to predict the KEGG functional profiling of microbial communities, and the analysis of functional profiling differences between various groups was performed using STAMP (v 2.1.3) software, as outlined in previous references [20,21].

### 2.9. Detection of SCFAs

GC-MS was used to analyze the SCFAs in the intestinal contents, following the methods outlined by Yan et al. [22]. A quantity of 100 mg of colon contents was diluted with 1 mL deionized water and mixed thoroughly in an iced water bath. Then, 200 μL of 50% sulfuric acid, 100 μL of 10 mg/L cyclohexanone solution, and 1 mL of ether were added and the mixture was homogenized for 1 min. Samples were centrifuged at 12,000 rpm for 10 min at 4℃, and the supernatants were obtained and analyzed.

### 2.10. Statistical Analysis

The data was analyzed by SPSS 24.0 software, with statistical significance set at *p* < 0.05. Graphs were created by GraphPad Prism 8.0 software.

## 3. Results

### 3.1. Effects of B. longum K5 in Alleviating Clinical Symptoms

**Figure 1:**
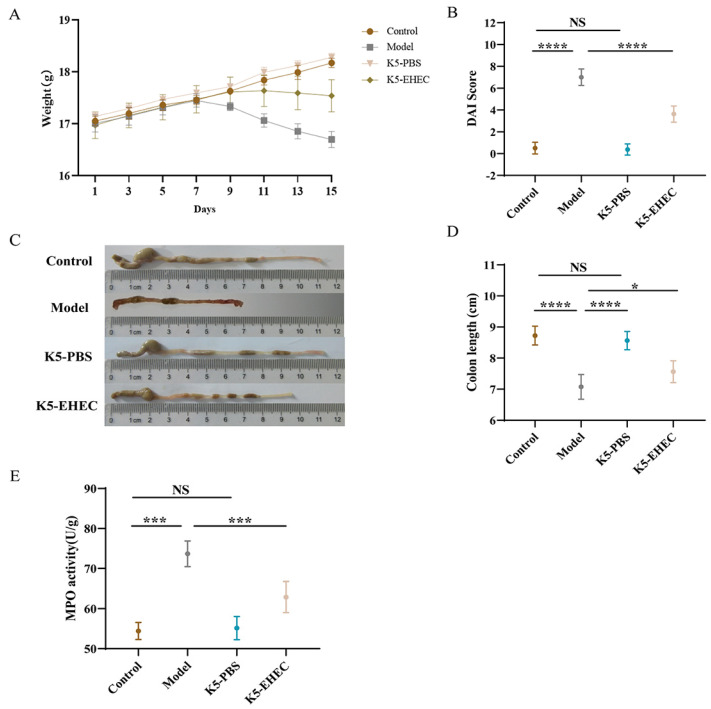
Effect of *B. longum* K5 on EHEC-induced clinical symptoms. Note: (**A**) changes in mouse body weight; (**B**) Disease Activity Index (DAI) scores; (**C**) representative images of mouse colons; (**D**) colon length; (**E**) MPO activity. Comparisons with different significance levels: (*) *p* < 0.05, (***) *p* < 0.001, and (****) *p* ≤ 0.0001. NS = not significant.

Initially, all the experimental groups of mice had identical body weights. However, after being administered EHEC O157:H7, the mice in the model and K5-EHEC groups experienced weight loss. The K5-EHEC group of mice had lesser weight loss when compared to the model group, indicating that *B. longum* K5 may be effective in protecting against the weight loss caused by EHEC O157:H7 (Figure 1A). In contrast to those in the control group, DAI scores in the model group showed a significant increase following EHEC O157:H7 infection (*p* < 0.0001) (Figure 1B). The K5-EHEC group markedly inhibited the elevation of DAI scores as compared to the model group (*p* < 0.0001). Additionally, EHEC O157:H7 infection caused a reduction in colon length in the mice, which was significantly lower in the model group in comparison to the control and K5-PBS groups (*p* < 0.0001). *B. longum* K5 prevented the shortening of the colon, with a significant increase observed in colon length in the K5-EHEC group compared to the model group (*p* < 0.05) (Figure 1C,D).

The level of neutrophil infiltration in response to inflammatory stimuli can be indicated by colonic MPO activity. The model group showed a significant elevation in colonic MPO activity when compared to the control group (*p* < 0.001). The activity of MPO in the K5-EHEC group was observed to be significantly lower than that in the model group (*p* < 0.001) (Figure 1E). Pretreatment with *B. longum* K5 halted the increase in MPO activity in the colon tissue of the mice.

### 3.2. Effects of B. longum K5 on Intestinal Pathological Changes

**Figure 2:**
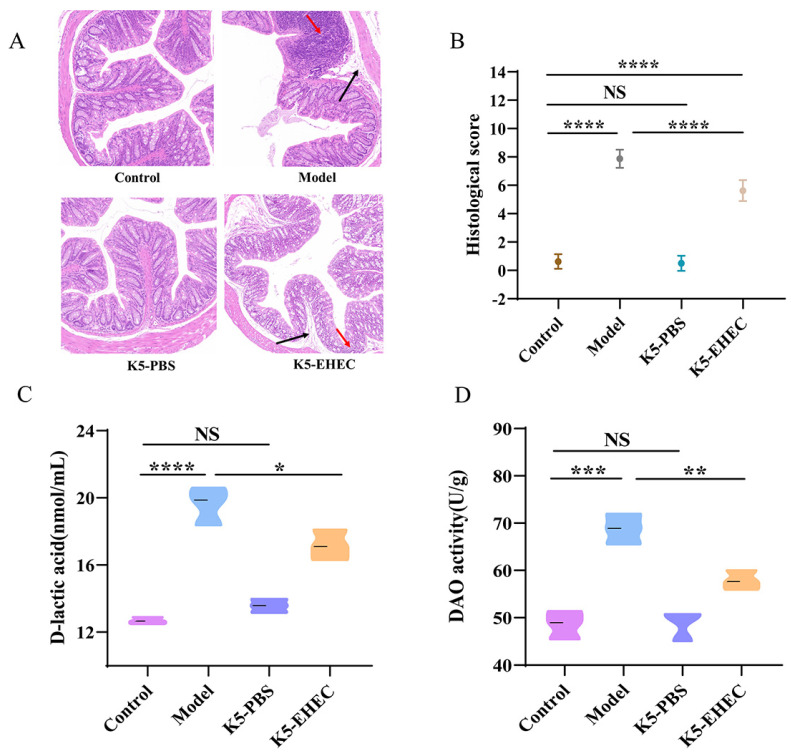
Effect of B. longum K5 on intestinal permeability. Note: (**A**) hematoxylin and eosin (H&E) staining (magnification ×100); Red arrows: inflammatory infiltration, Black arrows: increased tissue interstitial space (**B**) histological score; (**C**) D-lactic acid level; (**D**) DAO activity. Comparisons with different significance levels: (*) *p* < 0.05, (**) *p* < 0.01, (***) *p* < 0.001, and (****) *p* ≤ 0.0001. NS = not significant.

EHEC O157:H7 infection led to substantial colonic tissue damage. In the control group, mice displayed normal histological features in the colon. Conversely, the model group exhibited a significant deterioration in colonic epithelial integrity, marked by a reduction in goblet cell counts, expanded submucosal space, and widespread necrotic damage within the intestinal mucosa. In contrast to the model group, in the K5-EHEC group, the tissue damage caused by EHEC O157:H7 was alleviated and the major structures of colonic epithelial cells were preserved (Figure 2A).

The histopathological scores revealed a significant increase in colonic tissue scores in both the model and K5-EHEC groups following EHEC O157:H7 stimulation (*p* < 0.0001). The histopathological scores of the K5-EHEC group were lower than those of the model group after the *B. longum* K5 intervention (*p* < 0.0001) (Figure 2B). These findings indicate the efficacy of *B. longum* K5 in mitigating pathological damage to the gut in EHEC O157:H7- infected mice, thus safeguarding the integrity of the gut.

### 3.3. Effect of B. longum K5 on Intestinal Permeability

Serum D-lactate and DAO activity levels were used as markers to evaluate intestinal permeability. Comparative analysis revealed a marked increase in serum D-lactic acid levels and DAO activity in the model group compared to the control group (*p* < 0.001). Both the increases in serum D-lactate levels and DAO activity were effectively prevented in the K5-EHEC group compared to the model group (*p* < 0.05) (Figure 2C,D). These findings underscore the potential effectiveness of *B. longum* K5 in alleviating the increased intestinal permeability induced by EHEC O157:H7.

### 3.4. Effect of B. longum K5 on Gut Barrier Function

**Figure 3:**
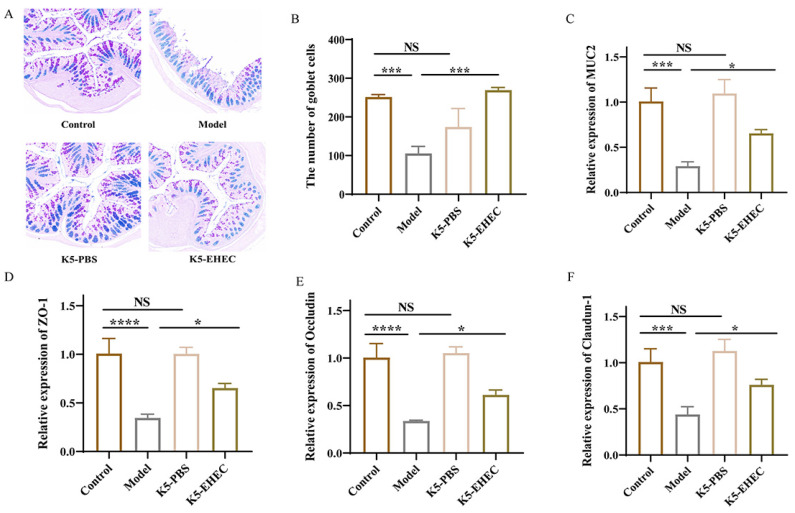
Effect of *B. longum* K5 on intestinal barrier function. Note: (**A**) AB-PAS staining of colon tissue sections (AB-PAS staining, magnification ×100); (**B**) number of goblet cells; (**C**) MUC2; (**D**) ZO-1; (**E**) Occludin; (**F**) Claudin-1. Comparisons with different significance levels: (*) *p* < 0.05, (***) *p* < 0.001, and (****) *p* ≤ 0.0001. NS = not significant.

AB-PAS staining was utilized to visualize goblet cells and mucin secretion in the gut. Colonic mucin secretion was diminished in the model mice compared to both the control and K5-PBS groups. The K5-EHEC group exhibited elevated levels of colonic mucin compared to the model group (Figure 3A). Additionally, the number of goblet cells in the model group was considerably less than that in the control group (*p* < 0.001). The K5-EHEC group exhibited a prevention of the reduction in the number of colonic goblet cells compared to the model group (*p* < 0.001) (Figure 3B).

Compared with that in the control group, the mRNA expression level of MUC2 in the colon tissue of the K5-PBS group was slightly increased, but the difference was not significant (*p* > 0.05). EHEC O157:H7 infection led to a significant decrease in the mRNA expression levels of MUC2 in the colons of mice in the model and K5-EHEC groups compared with the control group (*p* < 0.001). However, the K5-EHEC group prevented the reduction in MUC2 mRNA expression compared to the model group (*p* < 0.05) (Figure 3C). These findings indicate a reduction in MUC2 expression levels due to EHEC O157:H7 infection, whereas pretreatment with *B. longum* K5 protected intestinal epithelial cells and mitigated the decrease in MUC2 expression.

The expression levels of Occludin and Claudin-1 mRNA in the colonic tissues of mice in the K5-PBS group were slightly elevated when compared to those in the control group, but the difference was not significant (*p* > 0.05). The mRNA expression of tight junction proteins was significantly reduced in the colonic tissues of mice in the model group compared to the control group (*p* < 0.001). Notably, the K5-EHEC group effectively halted the decrease in mRNA expression levels of tight junction proteins compared with the model group (*p* < 0.05) (Figure 3D–F). These observations emphasize the effectiveness of pretreatment with *B. longum* K5 in mitigating the decrease in mRNA expression of tight junction proteins (ZO-1, Occludin, and Claudin-1) induced by EHEC O157:H7 infection in colonic tissues. This played a crucial role in reinforcing the function of the gut barrier.

### 3.5. Effect of B. longum K5 on Gut Inflammation

**Figure 4:**
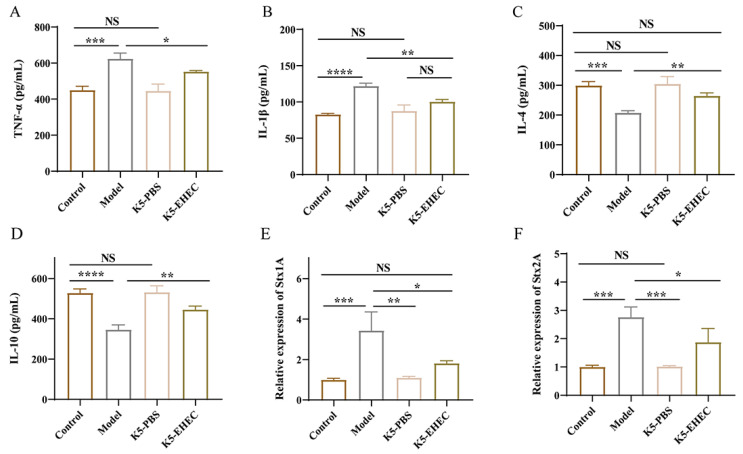
Effect of *B. longum* K5 on intestinal inflammation. Note: (**A**) TNF-α; (**B**) IL-1β; (**C**) IL-4; (**D**) IL-10; (**E**) Stx1A; (**F**) Stx2A. Comparisons with different significance levels: (*) *p* < 0.05, (**) *p* < 0.01, (***) *p* < 0.001, and (****) *p* ≤ 0.0001. NS = not significant.

The levels of TNF-α, IL-1β, IL-4, and IL-10 were measured in mouse colon tissue to reflect the extent of gut inflammation. Compared with those in the control group, the concentrations of TNF-α and IL-1β exhibited a marked increase in the colons of mice in the model group (*p* < 0.001). The concentrations of IL-4 and IL-10 in the colons of the model group were notably reduced compared to those of the control group (*p* < 0.001). As depicted in Figure 4A–D, the K5-EHEC group notably inhibited the elevation of TNF-α and IL-1β levels and prevented the reduction in IL-4 and IL-10 levels compared to the model group (*p* < 0.05). Remarkably, IL-1β levels in the K5-EHEC group did not show a significant difference compared to those in the K5-PBS group (*p* > 0.05). IL-4 levels in the K5-EHEC group were comparable to those in the control group (*p* > 0.05). These findings emphasize the ability of *B. longum* K5 to mitigate the inflammatory response induced by EHEC O157:H7 infection through the inhibition of pro-inflammatory cytokine production and the promotion of anti-inflammatory cytokine production.

### 3.6. Effect of B. longum K5 on the Expression of Virulence Factors

The analysis of cecal contents from the mice in the model group revealed a substantial increase in the mRNA expression levels of Stx1A and Stx2A compared to the control and K5-PBS groups (*p* < 0.01). This trend, however, was reversed in the K5-EHEC group, where a significant suppression of Stx1A and Stx2A expression was observed (*p* < 0.05), as detailed in Figure 4E,F. Remarkably, the mRNA expression level of Stx1A in the K5-EHEC group did not exhibit a significant deviation from that of the control group (*p* > 0.05). This implies that pretreatment with *B. longum* K5 alleviated the elevated expression levels of Stx1A and Stx2A induced by EHEC O157:H7 infection, underscoring the role of *B. longum* K5 in mitigating the effects of this infection.

### 3.7. Effect of B. longum K5 on the Diversity of the Gut Microbiota

**Figure 5:**
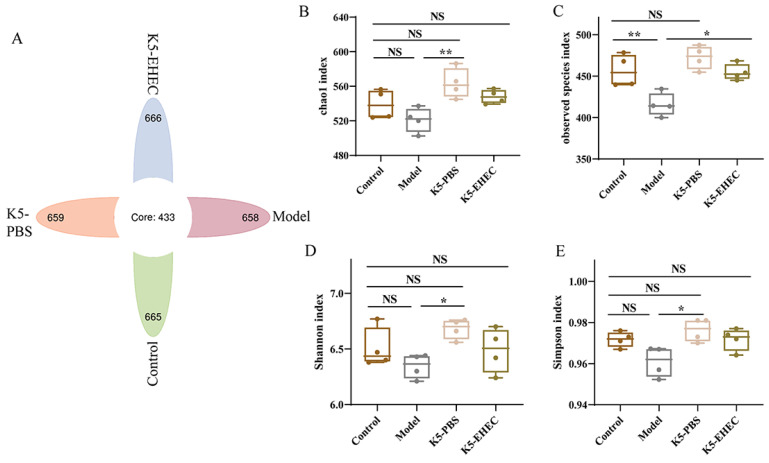
Effect of *B. longum* K5 on intestinal flora diversity. Note: (**A**) OTU flower plot; (**B**) Chao1 index; (**C**) observed species index; (**D**) Shannon index; (**E**) Simpson index. Comparisons with different significance levels: (*) *p* < 0.05, (**) *p* < 0.01. NS = not significant.

The impact of *B. longum* K5 on the gut microbiota was investigated through 16S rRNA sequencing. The flower plot results revealed that the unique operational taxonomic unit (OTU) counts for the control, model, K5-PBS, and K5-EHEC groups were 665, 658, 666, and 659, respectively (Figure 5A). The Chao1, observed species, Shannon, and Simpson indices were used to evaluate the diversity of the gut microbiota. There was no significant difference in species richness and diversity between the K5-PBS group and the control group (*p* > 0.05). A marked decrease in the observed species index was observed in the model group compared to the control group (*p* < 0.01). However, the K5-EHEC group mitigated this significant decrease in the observed species index compared to the model group (*p* < 0.05), and the Chao1, Shannon, and Simpson indices of the K5-EHEC group were not significantly different from those of the control group (*p* > 0.05) (Figure 5B–E). These findings indicate that EHEC O157:H7 infection diminished the gut microbial diversity in the mice, while pretreatment with *B. longum* K5 appeared to prevent the reduction in microbial diversity in the gut environment caused by EHEC O157:H7 infection.

### 3.8. Effect of B. longum K5 on the Structure of the Gut Microbiota

**Figure 6:**
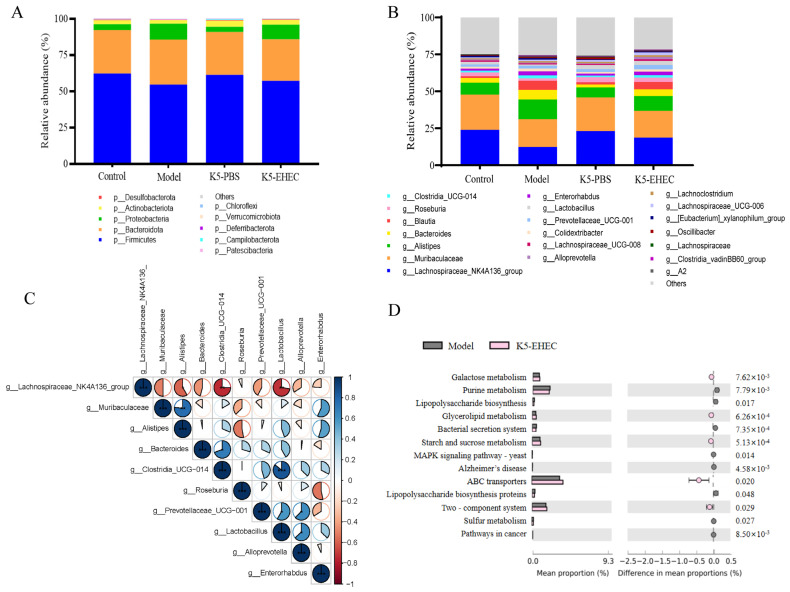
Effect of *B. longum* K5 on intestinal microflora structure and microbiota functional prediction using PICRUSt. Note: (**A**) phylum level; (**B**) genus level; (**C**) correlation analysis of gut microbial alteration; (**D**) prediction of the gut microbiota function. Comparisons with different significance levels: (*) *p* < 0.05, (**) *p* < 0.01, (***) *p* < 0.001.

In this study, we examined the effect of EHEC O157:H7 infection on the intestinal microbial composition, focusing on both the phylum and genus levels. In the control and K5-PBS groups, the gut microbiota predominantly comprised *Firmicutes*, *Bacteroidota*, and *Proteobacteria*. Notably, the K5-PBS group exhibited a marginal rise in *Actinobacteriota*. The model group had a reduction in the abundance of *Firmicutes* and *Bacteroidota*, accompanied by a surge in *Proteobacteria*. Conversely, pretreatment with K5 in the K5-EHEC group lessened the increase in *Proteobacteria* and the decrease in *Firmicutes* (Figure 6A). At the genus level, the model group demonstrated an elevation in the relative abundance of *Alistipes*, *Bacteroides*, *Blautia*, and *Desulfovibrio* following infection with EHEC O157:H7. Meanwhile, the relative abundance of the *Lachnospiraceae* NK4A136 group, *Muribaculaceae*, *Roseburia*, and *Lactobacillus* decreased in the model group. These findings demonstrate that pretreatment with *B. longum* K5 effectively prevented the decrease in abundance of the *Lachnospiraceae* NK4A136 group, *Roseburia*, and *Lactobacillus* while inhibiting the proliferation of *Alistipes* associated with EHEC O157:H7 infection. There was a slight increase in the abundance of *Lactobacillus* in the K5-PBS group compared to the control group (Figure 6B).

Correlation analyses of the primary genera within the gut microorganisms of K5-EHEC demonstrated a notably negative correlation between the levels of *Clostridia* UCG-014 and *Lactobacillus* and the levels of *Lachnospiraceae* NK4A136 (*p* < 0.01). *Lactobacillus* and *Clostridia* UCG-014 exhibited a significantly positive correlation with each other (*p* < 0.001); a highly positive correlation was also observed between *Alistipes* and *Muribaculaceae* (*p* < 0.01) (Figure 6C). This indicates that EHEC O157:H7 disrupted the typical composition of the gut microbiota in mice, whereas *B. longum* K5 seems to have reduced the induction of this effect by promoting the growth of beneficial microbiota. The functional prediction of the KEGG pathway indicated that *B. longum* K5 prevented the decrease in the abundance of microbial genes related to galactose, glycerolipid, starch, and sucrose metabolism induced by EHEC O157:H7 infection. Treatment with *B. longum* K5 concurrently halted the downregulation of genes associated with ABC transporter proteins and the two-component system. On the contrary, the increase in genes associated with the bacterial secretion system, sulfur metabolism, lipopolysaccharide biosynthesis proteins, and pathways related to Alzheimer’s disease, the MAPK signaling pathway, and cancer was significantly inhibited (Figure 6D).

### 3.9. Production of SCFAs

**Figure 7:**
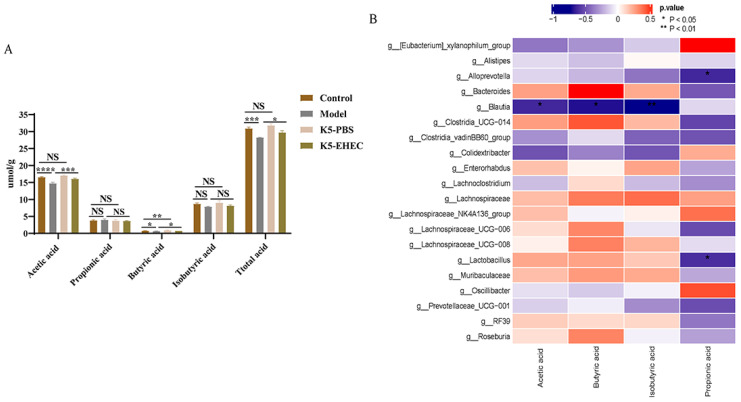
Levels of short-chain fatty acids in intestinal contents. Note: (**A**) short-chain fatty acids; (**B**) heatmap diagram of the correlations between SCFAs and genera with high abundance in the gut microbiota. Comparisons with different significance levels: (*) *p* < 0.05, (**) *p* < 0.01, (***) *p* < 0.001, and (****) *p* ≤ 0.0001. NS = not significant.

The results of SCFAs in the intestinal contents of the mice revealed a marginal increase in the levels of acetic, isobutyric, and total acids in the K5-PBS group compared to the control group. However, the difference was not statistically significant (*p* > 0.05). The concentrations of butyric acid were significantly higher in the K5-PBS group compared to the control group (*p* < 0.01). And the model group exhibited the lowest levels of acetic acid, butyric acid, and total acid. Remarkably, pretreatment with *B. longum* K5 in the K5-EHEC group significantly promoted butyric acid and total acid production as compared to the model group (*p* < 0.05) (Figure 7A).

A correlation analysis of SCFAs with the main genera of gut microorganisms showed that *Alloprevotella* and *Lactobacillus* were significantly and negatively correlated with the propionic acid content, and *Blautia* was significantly and negatively correlated with acetic, butyric, and isobutyric acids (*p* < 0.05) (Figure 7B). *B. longum* K5 demonstrated the potential to promote the production of SCFAs, particularly butyric acid, in the intestinal contents of mice.

## 4. Discussion

EHEC O157:H7 is considered a highly dangerous foodborne pathogen capable of colonizing the epithelial surface of the intestinal mucosa [23,24], forming A/E lesions, and impairing the function of the intestinal epithelial barrier [25]. Stx produced by EHEC O157:H7 can be transmitted into the bloodstream, causing toxemia and extensive histopathological damage and increasing the risk of HUC [26]. Probiotics have garnered significant attention for their potential role in preventing pathogenic infections. A study conducted by Wang et al. revealed that *L. casei* LC2W effectively inhibited the intestinal colonization of EHEC O157:H7 by directly or indirectly antagonizing the bacterial association with epithelial cells [27]. The fermented broth of *L. acidophilus* has been demonstrated to prevent the intestinal barrier dysfunction induced by EHEC O157:H7 [28]. *B. longum* has been reported to have health-promoting benefits of inhibiting harmful intestinal bacteria [29], lowering cholesterol levels [30], modulating the gut microbiota, and enhancing immune response [16,31]. Bifidobacterium is known to secrete various antimicrobial agents, including organic acids and bacteriocins [32,33]. Moreover, the mechanisms through which *Bifidobacteria* exert their antibacterial effects include suppressing the expression of virulence factors, competing for nutrients, and obstructing the adhesion and invasion of pathogenic bacteria to host cells by contending for binding sites [34]. Therefore, our research utilized a previously established EHEC O157:H7-infected mouse model [35] to explore the protective effects of *B. longum* K5 and to elucidate the mechanism of action.

The experimental group of mice exhibited symptoms such as diarrhea, shortened colons, weight loss, and intestinal wall thinning after one week of gavage with EHEC O157:H7, confirming the effective establishment of the EHEC O157:H7 infection model in comparison to the control group. Mice in the K5-EHEC group pre-supplemented with *B. longum* K5 showed mitigation of these symptoms, including the weight loss and shortened colon. The increase in the DAI score further indicated the efficacy of *B. longum* K5 in alleviating infection symptoms in the mice. These observations were in agreement with the study by Gagnon et al., where *B. thermophilum* RBL 71 enhanced food intake and substantially reduced weight loss in infected mice [36]. Our research showed that introducing *B. longum* K5 effectively attenuated the EHEC O157:H7-induced rise in MPO activity, suggesting a reduction in neutrophil infiltration. This aligns with the findings reported by Wang et al. [27], emphasizing the potential therapeutic role of *B. longum* K5 in controlling inflammation.

The structural integrity of the intestinal epithelium is crucial in resisting pathogenic bacterial invasion [37]. In our study, EHEC O157:H7 disrupted this integrity in mice, evidenced by damage to the intestinal epithelial microvilli and mucosal lining. Conversely, mice treated with *B. longum* K5 showed attenuated intestinal damage, less severe inflammatory infiltration, better-preserved intestinal epithelial structures, and significantly improved colon tissue pathology, corroborating the findings of Wang et al. [32]. The functionality of the gut barrier is connected to numerous clinical conditions, including bacterial infections [38]. Pathogenic organisms can undermine this barrier by altering the expression of TJ proteins and producing toxins, thereby causing cell damage, apoptosis, and heightened permeability in intestinal epithelial cells [39]. EHEC O157:H7 infection significantly reduced the mRNA expression of Claudin-1, Occludin, and ZO-1. And there was a notable increase in serum DAO and D-lactic acid levels. Contrastingly, *B. longum* K5 treatment effectively counteracted these changes. This aligns with Bao et al.’s observations, wherein *Bacillus amyloliquefaciens* TL106 was found to mitigate TJ protein expression reduction in the colon and decrease DAO and D-lactic acid levels in serum [40]. Mucins, serving as initial barriers on cell surfaces, play a pivotal role in hindering the invasion of pathogenic microorganisms [41]. MUC2, predominantly expressed in goblet cells, is a critical component of the mucus layer, essential for maintaining intestinal balance [41,42]. The pathogen EHEC O157:H7 can degrade mucin via its StcE secretion, while probiotics are known to promote MUC2 production in goblet cells, thereby reinforcing the gut barrier [43,44]. Our experiment revealed that pre-supplementation of mice with *B. longum* K5 prevented the EHEC O157:H7-induced decrease in MUC2 mRNA expression and resulted in less damage to goblet cells. This is consistent with the findings of Wang et al., who reported that *Lactobacillus casei* LC2W can bolster the colonic mucosal layer by enhancing MUC2 expression [45].

The balance between pro-inflammatory and anti-inflammatory cytokines determines the inflammation outcome during bacterial infections [46,47]. Consistent with prior findings [27,48], our study observed heightened levels of pro-inflammatory cytokines TNF-α and IL-1β in the colons of mice infected with EHEC O157:H7. Notably, the pretreatment with *B. longum* K5 prevented these elevations in the concentrations of TNF-α and IL-1β in the colon tissue. Key anti-inflammatory cytokines like IL-4 and IL-10 are essential in modulating inflammatory responses. EHEC O157:H7 infection led to a significant diminishment of anti-inflammatory factors in colon tissue, a trend that was notably prevented in mice treated with *B. longum* K5. This aligns with the observations of Bao et al. [40], who reported that *Bacillus amylolyticus* TL106 curbed the production of pro-inflammatory factors like TNF-α, IL-1β, IL-6, and IL-8 and mitigated the decline in IL-10 levels in the intestinal tissues of mice infected with EHEC O157:H7. Stx1A and Stx2A are key virulence factors in EHEC O157:H7, playing a pivotal role in the development of hemorrhagic diarrhea and HUC by disrupting protein synthesis in eukaryotic cells. Research by Yoshimura et al. highlighted that certain strains of *Bifidobacteria* can markedly lower Shiga toxin levels in both the cecal contents and serum of mice [11]. In line with these findings, our study also observed that the elevated levels of Stx1A and Stx2A resulting from EHEC O157:H7 infection were inhibited in both the serum and cecal contents of mice pretreated with *B. longum* K5. Furthermore, a study by Saito et al. involved pre-inoculating mice with *Bacteroides fragilis* and *vulgatus* before EHEC O157:H7 infection. Their results showed a notable reduction in Stx1 and Stx2 levels and a significant decrease in mortality rates compared to those in mice infected only with EHEC O157:H7 [49].

The gut microbiota, nestled within the mucous layer, acts as a natural defense, curbing the proliferation of pathogenic bacteria. This is achieved through various mechanisms, such as competing for colonization sites and nutrients and secreting antimicrobial agents [50]. A balanced gut microbiota is critical in modulating the host’s immune response, thereby reducing the inflammatory response induced by bacterial infections [51]. In this study, we found that *B. longum* K5 was effective in preventing the loss of microbial diversity in the gut microbiota of mice caused by EHEC O157:H7 infection. Mice treated with *B. longum* K5 exhibited a gut microbiota structure similar to that in the control group. At the genus level, a decrease in the relative abundance of the *Lachnospiraceae* NK4A136 group, *Muribaculaceae*, and *Roseburia* was observed following EHEC O157:H7 infection, while *B. longum* K5 administration prevented the decrease in these genera. *B. longum* K5 also raised the relative abundance of *Roseburia*, *Lactobacillus*, and *Oscillibacter*. These observations align with the findings of Hu et al., wherein *L. johnsonii*, *L. plantarum*, and *L. rhamnosus* were shown to elevate the relative abundance of *Lachnospira*, *Ruminococcus*, *Roseburia*, and *Blautia* in the intestine of EHEC-infected mice [52]. These findings suggest that early *B. longum* K5 intervention can bolster gut microbiota diversity, counteracting the microbial dysbiosis induced by EHEC O157:H7 infection. PICRUSt predictions indicated that the model group, infected with EHEC O157:H7, showed an upregulation of genes linked to lipopolysaccharide biosynthesis. In contrast, the increase in these genes was prevented in the K5-EHEC group. This pattern is consistent with our observation that *B. longum* K5 substantially mitigated the levels of inflammatory cytokines. The model group infected with EHEC O157:H7 demonstrated an elevated gene abundance in sulfur metabolism. This aligns with our earlier observation of an increased relative abundance of *Desulfovibrio* in the gut microbiota of these mice.

SCFAs, including acetic acid, propionic acid, and butyric acid, are the primary products of complex nutrient fermentation by the gut microbiota [53]. SCFAs play a multifaceted role: they serve as energy sources for intestinal epithelial cells and influence the expression of virulence genes in pathogenic bacteria, thereby impacting their cellular metabolism and pathogenicity [54,55]. In mice infected with EHEC O157:H7, we observed a significant alteration in SCFA production. There was a substantial decrease in the total acid content, with both the acetic acid and butyric acid levels dropping below those in the control group. However, treatment with *B. longum* K5 mitigated these changes. This aligns with prior research showing elevated SCFA concentrations in the fecal samples of mice treated with *Bifidobacterium* [56]. Moreover, our findings are consistent with those of Fukuda et al., who demonstrated that *Bifidobacterium*, through acetate production, can hinder the transport of Shiga toxins from the intestinal tract to the bloodstream, thereby offering protection against EHEC O157:H7-induced mortality in mice [8]. Indeed, in the event of an EHEC epidemic, pre-treatment with probiotics, such as *Bifidobacterium*, could be considered for individuals at heightened risk of infection or complications. This approach may involve providing prophylactic *Bifidobacterium* supplementation to vulnerable populations, such as individuals with weakened immune systems, young children, or the elderly. Prophylactic *Bifidobacterium* supplementation could contribute to the management and control of the epidemic by bolstering gut health and potentially reducing the susceptibility to EHEC infection or mitigating its severity.

## 5. Conclusions

In summary, our research demonstrated the potential protective effects of *B. longum* K5 against EHEC O157:H7 infection in mice. *B. longum* K5 prevented EHEC O157:H7-induced weakening of the gut barrier, preserved the abundance of beneficial microbes, and promoted the production of SCFAs in the colons of the mice. Moreover, it inhibited the increase in the concentration of pro-inflammatory cytokines and pathogenic virulence factors. These insights underscore the potential of *B. longum* K5 as a novel therapeutic agent in preventing infections induced by EHEC O157:H7.

## Figures and Tables

**Table 1:** Experimental design for the animal model.

Group	−6–0 d	1–7 d	7–14 d
Control	Diet ad libitum	0.2 mL PBS	0.2 mL PBS
Model	Diet ad libitum	0.2 mL PBS	0.2 mL EHEC O157:H7
K5-PBS	Diet ad libitum	0.2 mL *B. longum* K5	0.2 mL PBS
K5-EHEC	Diet ad libitum	0.2 mL *B. longum* K5	0.2 mL EHEC O157:H7

## Data Availability

The data presented in this study are available from the author upon reasonable request.

## Supplementary Materials

The following supporting information can be downloaded at: https://www.mdpi.com/article/10.3390/nu16081164/s1. Table S1: Disease Activity Index (DAI) Scoring System. Table S2: Histopathological Scoring System. Table S3: Primer Sequences for RT-qPCR Assays in Colonic Tissue.
