# Supplementary material for: Bifidobacterium longum K5 Prevents Enterohaemorrhagic Escherichia coli O157:H7 Infection in Mice through the Modulation of the Gut Microbiota
Source: Nutrients. 2024 Apr 13;16(8):1164. doi: 10.3390/nu16081164 (PMC11053520; doi:10.3390/nu16081164)
Supplement: Supplementary file 1 [file nutrients-16-01164-s001.zip › nutrients-2950804-supplementary.pdf]

Supplementary Materials

Table S1: Disease Activity Index (DAI) Scoring System

Score	weight loss (%)	stool consistency	occult/gross bleeding
0	none	normal	normal
1	0-5		
2	5-10	loose stools	hemoccult positive
3	10-20		
4	> 20	diarrhea	gross bleeding

Table S2: Histopathological Scoring System

Score	inflammatory infiltrate	mucosal damage	crypt damage
0	none	none	none
1	mild	mucosa	1/3 damaged
2	moderate	mucosa and submucosa	2/3 damaged
3	severe	transmural	crypt loss, but surface epithelium present
4			Both crypt and surface epithelium were lost.

Table S3: Primer Sequences for RT-qPCR Assays in Colonic Tissue

Gene	Forward (5′–3′)	Reverse (5′–3′)
Muc2	CGAGCACATCACCTACCACATCATC	TCCAGAATCCAGCCAGCCAGTC
ZO-1	AACCCGAAACTGATGCTGTGGATAG	CGCCCTTGGAATGTATGTGGAGAG
Occludin	TTGGCTACGGAGGTGGCTATGG	CCTTTGGCTGCTCTTGGGTCTG
Claudin-1	GCTGGGTTTCATCCTGGCTTCTC	CCTGAGCGGTCACGATGTTGTC
Stx1A	CCATTCTGGCAACTCGCG	GGCAAGAGCGATGTTACGGT
Stx2A	TTGCTGTGGATATACGAGGGC	TCCGTTGTCATGGAAACCG
Gapdh	GACAGCCGCATCTTCTTGTG	AATCCGTTCACACCGACCTT
